# Inter-Device Agreement between Fitbit Flex 1 and 2 for Assessing Sedentary Behavior and Physical Activity

**DOI:** 10.3390/ijerph18052716

**Published:** 2021-03-08

**Authors:** Sunku Kwon, Ryan D. Burns, Youngwon Kim, Yang Bai, Wonwoo Byun

**Affiliations:** 1 Department of Health and Kinesiology, University of Utah, Salt Lake City, UT 84112, USA; sunku.kwon@utah.edu (S.K.); ryan.d.burns@utah.edu (R.D.B.); yang.bai@utah.edu (Y.B.); 2 School of Public Health, Li Ka Shing Faculty of Medicine, The University of Hong Kong, Hong Kong; youngwon.kim@hku.hk; 3 MRC Epidemiology Unit, School of Clinical Medicine, University of Cambridge, Cambridge CB2 0SL, UK

**Keywords:** sedentary behavior, physical activity, inter-device agreement, epidemiological

## Abstract

This study examined the inter-model agreement between the Fitbit Flex (FF) and FF2 in estimating sedentary behavior (SED) and physical activity (PA) during a free-living condition. 33 healthy adults wore the FF and FF2 on non-dominant wrist for 14 consecutive days. After excluding sleep and non-wear time, data from the FF and FF2 was converted to the time spent (min/day) in SED and PA using a proprietary algorithm. Pearson’s correlation was used to evaluate the association between the estimates from FF and FF2. Mean absolute percent errors (MAPE) were used to examine differences and measurement agreement in SED and PA estimates between FF and FF2. Bland-Altman (BA) plots were used to examine systematic bias between two devices. Equivalence testing was conducted to examine the equivalence between the FF and FF2. The FF2 had strong correlations with the FF in estimating SED and PA times. Compared to the FF, the FF2 yielded similar SED and PA estimates along with relatively low measurement discords and did not have significant systematic biases for SED and Moderate-to-vigorous PA estimates. Our findings suggest that researchers may choose FF2 as a measurement of SED and PA when FF is not available in the market during the longitudinal PA research.

## 1. Introduction

It is no secret that regular physical activity (PA) provides many health benefits. Benefits include, but are not limited to, reduced hypertension, obesity, type-2 diabetes, as well as lower medical costs across the lifespan [1,2,3]. Objective and accurate self-monitoring of PA can effectively facilitate the engagement of adequate amounts and intensities [4]. Recently, consumer-based activity monitors, like Fitbit, have been widely used for assisting individuals’ self-monitoring of PA and sleep. These activity monitors track time spent in PA and sedentary behavior (SED) and, energy expenditure (EE), and daily steps with efficiency and relatively low cost [5,6,7,8]. Built-in accelerometers in consumer activity monitors can estimate the frequency and intensity of the user’s PA by calculating the acceleration of the user’s bodily movement primarily based on the proprietary algorithm [9,10] Furthermore, most consumer activity monitors are typically worn on the wrist and connected to a mobile application, which increase users’ adherence to wearing time. With their popularity, capability, and cost-efficiency, consumer-based activity monitors have great potential for being effective tools that assess SED and PA in large cohorts within both clinical and research settings.

Fitbit (San Francisco, CA, USA) is a leading manufacturer of consumer-based activity monitors in the health and fitness market. Recent data showed that there were more than 29 million active Fitbit users in 2019 [11]. Fitbit devices have been increasingly used by researchers as a measurement or intervention tool of PA due to its inexpensive cost, real-time tracking, user-friendly dashboard and app, and its validity and reliability for estimating EE, step counts, activity intensity, sleep quality, and other personal metrics (e.g., socialization features) [8,12,13,14,15,16,17,18]. The Fitbit Flex™ (FF) is a wrist-worn activity monitor. It has been a popular model due to its competitive cost and simplicity of use. Recent studies have reported FF could provide comparable estimates of total EE, SED and total PA (TPA) time compared with other objective measures, such as research-based accelerometers, indirect calorimetry, and direct observation [5,19,20]. The previous study by Bai et al., showed that the FF similarly estimated total EE (mean bias: 20.4 kcal (6% difference)) compared with indirect calorimetry during an 80-min protocol of semi-structured activities [5]. Additionally, Redenius et al., reported that the FF had an acceptable measurement error (6.8% mean absolute percent error) in estimating SED compare to ActiGraph GT3X+ accelerometer under free-living condition [20]. Another study revealed that the FF accurately classified TPA in comparison with direct observation in children (area under the receiver operating curve: 0.92) [19]. These results indicate that FF is capable of being an assessment tool for PA surveillance in clinical and epidemiological research.

Despite the advantageous features of Fitbit, the manufacturer’s frequent updates may cause variation in its measurement properties. Due to the nature of the competitive commercial market, Fitbits updates occur much more frequently than those of research-grade accelerometers. Nevertheless, the National Institute of Health included Fitbit as a device of choice for a recently launched large scale longitudinal study (target N ≥ 1 million) called All of Us Research Program. This study aims to improve health and prevent disease using the data available via wearable technologies such as Fitbit. In the All of Us Research Program, participants provide the data collected by Fitbit to the study regardless of the model of Fitbit they use. This suggests the shorter lifespan of Fitbit models may not limit their utilization in longitudinal studies if the inter-device reliability is high across model generations.

The Fitbit Flex™ 2 (FF2) is the FF’s second generation with some obvious upgrades. The FF2 has improved battery life and is 30% lighter and smaller than the FF. Additionally, FF2 has a waterproof feature, which improves usability. Although the manufacturer has stated improved features for the FF2, the consistency between the FF and FF2 in activity monitoring, specifically the estimates of SED and PA, have not yet been examined by data-driven research. Therefore, the primary purpose of this study was to examine the inter-device agreement between the FF and FF2 for estimating time spent SED and PA in free-living conditions. The outcomes of this study will provide information for the potential cross-utilization of the FF and FF2 in PA surveillance and epidemiological research.

## 2. Materials and Methods

### 2.1. Participants

A non-probability convenience sample of 33 adult volunteers participated in this study. We excluded any participants who were under 18 years-old, physically disabled, pregnant, or unable to participate in regular PA as recommended by a physician associated with this study. This study was approved by the University Institutional Review Board, and all participants provided written consent to participate prior to data collection.

### 2.2. Instruments

The FF and the FF 2 (Fitbit, Inc., San Francisco, CA, USA) were used in this study. Both devices are light (FF: 14.6 g and FF2: 10.2 g), small (FF: 2.09 × 0.14 cm and FF2: 2.05 × 0.1 cm, including wristband) consumer-based and wrist-worn wearable activity monitors. A built-in triaxial accelerometer measures user’s PA characteristics such as activity intensity throughout the day. Both devices regularly sync with the Fitbit dashboard via Fitbit mobile application. Fitbit’s dashboard on the app shows user activity progress, exercise history, and sleep patterns. Additionally, both devices can record and store data, including steps, activity minutes, distance, EE, exercise, and sleep, for approximately seven days without syncing with the Fitbit dashboard. Users’ PA data recorded by both devices is transferred to the Fitbit application program interface (API) called Fitabase (Small Steps Lab, San Diego, CA, USA). Fitabase is a research application of the Fitbit devices and provides data from multiple Fitbit activity monitors in real-time.

### 2.3. Procedures

Prior to the data collection, the research staff explained the study protocol, and participants completed the informed consent form. Demographic information was obtained from participants at the beginning of the data collection session by a self-reported questionnaire. Once participants completed the demographic questionnaire, research staff measured the anthropometric characteristics of each participant. Height, weight, and waist circumference were measured using a stadiometer (Shorr Productions; Olney, MD, USA), an electric weight-scale (Seca, Model 770; Hamburg, Germany), and a tape measure (Gulick II, Gays Mills, WI, USA), respectively. To avoid measurement error, the research staff measured all the anthropometric characteristics three times. Body Mass Index (BMI; kg/m^2^) was calculated based on the measured height and weight. Following the anthropometric measures, participants were instructed how to properly wear two activity monitors (i.e., FF and FF2), then wore both devices on the non-dominant wrist for 14 consecutive days except while charging the devices and performing aquatic activities. All participants switched the wearing positions of the FF and FF2 (i.e., top and bottom of the wrist) when both devices had to be removed and worn during the data collection period. Throughout the 14-day period, participants recorded time when they removed devices and time when they slept using an Activity/Sleep Log Sheet.

### 2.4. Data Processing

Once participants completed the 14 days of instructed wear time, the activity intensity data measured by FF and FF2 were downloaded via the Fitabase. Using Fitbit’s proprietary algorithm, the acceleration data recorded by the FF and FF2 is converted into activity counts (i.e., SED = 0, light PA (LPA) = 1, Moderate PA (MPA) = 2, and Vigorous PA (VPA) = 3). The activity count data was then exported via Fitabase in 60-s sampling intervals (epochs) for the subsequent data processing. The activity counts data of the FF and FF2 were merged and aligned by participant ID, date, and time into a single dataset. The non-wear time and sleep time were excluded from the single dataset using the self-reported activity/sleep log. The current study only included the data with the valid time during waking hours of each day for statistical analyses. 

### 2.5. Statistical Analysis

Pearson product-moment correlation was used to determine the linear relationship between FF and FF2 in the overall estimation of SED and PA. Additionally, mean absolute percent errors (MAPEs) were calculated to determine the degree of FF2’s measurement agreements for SED and PA estimates compared to the FF. Bland-Altman (BA) Plots were used to present the agreement between FF and FF2 as well as evaluate any potential systematic biases in SED and PA. Pitman’s Tests for the difference between two correlated variances were performed to determine the equality of the variances in SED and PAs estimates between the two devices. To determine the equivalence between FF and FF2, this study performed equivalence tests. Ultimately, the equivalence was determined if the 90% CIs of the FF2’s SED and PA estimates fall within the ±10% equivalence zone (EZ) of the estimates from the FF. However, there is no evidence of the universal acceptable EZ range of the FF. Thus, the EZs were increased by 5% (i.e., 15%, 20%, and 25%) until equivalence was reached as well as we calculated the exact ranges of EZ that allows accepting the equivalence between FF and FF2. Data were analyzed using Stata 14 software (StataCorp LLC, College Station, TX, USA) and SAS 9.4 software (SAS Institute, Cary, NC, USA). Statistical significance was determined at *p* < 0.05.

## 3. Results

The mean age (±SD) of participants was 23.9 ± 0.8 years. There was no significant difference in age and wear time between males and females (Age: *p* = 0.28; Wear time: *p* = 0.33). Body mass index (BMI) for males and females were 27.8 ± 3.9 kg/m^2^ and 22.6 ± 2.2 kg/m^2^, respectively. Participants’ demographic information is summarized in Table 1.

**Figure 1:**
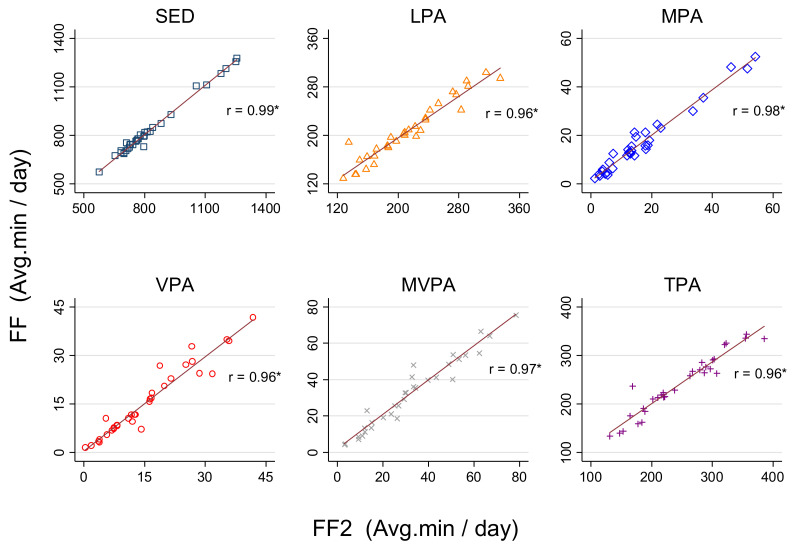
Relationship between FF and FF2 in estimating time spent in SED and PA. SED: Sedentary behavior; LPA: Light physical activity; MPA: Moderate physical activity; VPA: Vigorous physical activity, MVPA: Moderate-to-vigorous physical activity; TPA: Total physical activity; *: *p* < 0.05.

We found very strong correlations (r > 0.95, *p* < 0.01) for SED and PA estimates between FF and FF2 (Figure 1).

The mean difference and MAPE values of SED and PA estimates between FF and FF2 are presented in Table 2. In general, the FF2 slightly underestimated SED time (mean difference: 6 min/day; 95% CI: −0.7 to 12.7) and overestimated LPA time (mean difference: −6.3 min/day; 95% CI: −11.8 to −0.7). The values of MAPE between FF and FF2 were relatively small (MAPE range: 0.1%–3.1%).

**Figure 2:**
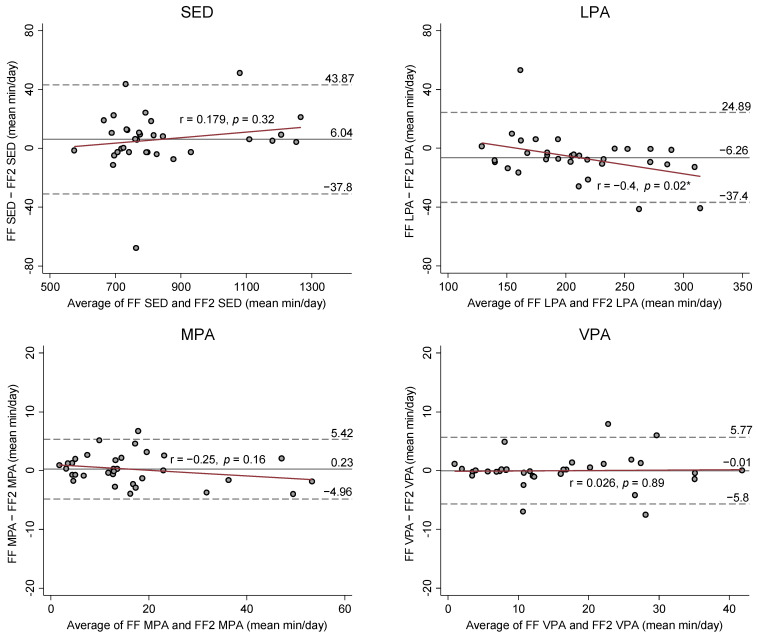

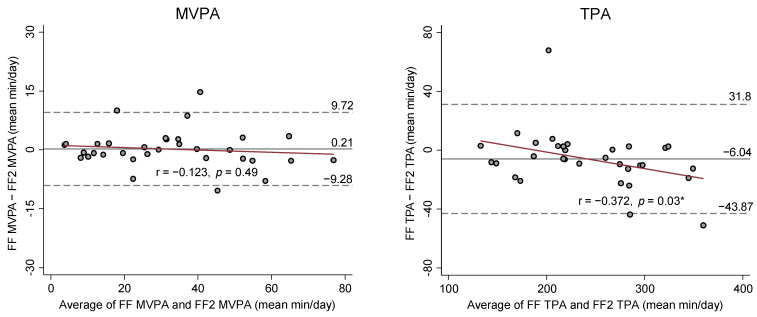
Bland-Altman plot comparing estimates of SED and PA between FF and FF2. Dashed lines show 95% limits of agreement (±1.96 standard deviation); SED: Sedentary behavior; LPA: Light physical activity; MPA: Moderate physical activity; VPA: Vigorous physical activity, MVPA: Moderate-to-vigorous physical activity; TPA: Total physical activity; *: *p* < 0.05.

Figure 2 presents Bland-Altman (BA) Plots on the mean differences and 95% limits of agreements between SED and PA estimates from the FF and FF2. BA plots showed no apparent bias for the agreement and variance in SED and PA estimates between FF and FF2, except for LPA and TPA estimates. The results from the Pitman’s Test were r = −0.4 (*p* = 0.02) for LPA and r = −0.37 (*p* = 0.03) for TPA, indicating that the FF2 increasingly overestimated LPA and TPA compared to the FF as mean volume of LPA and TPA increased.

**Figure 3:**
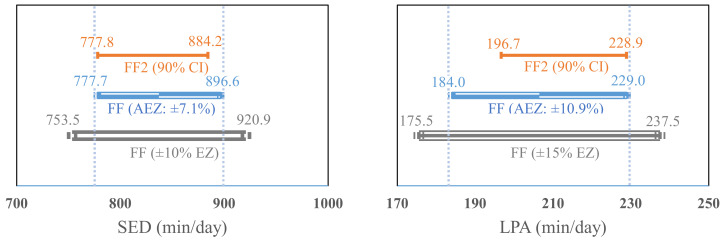

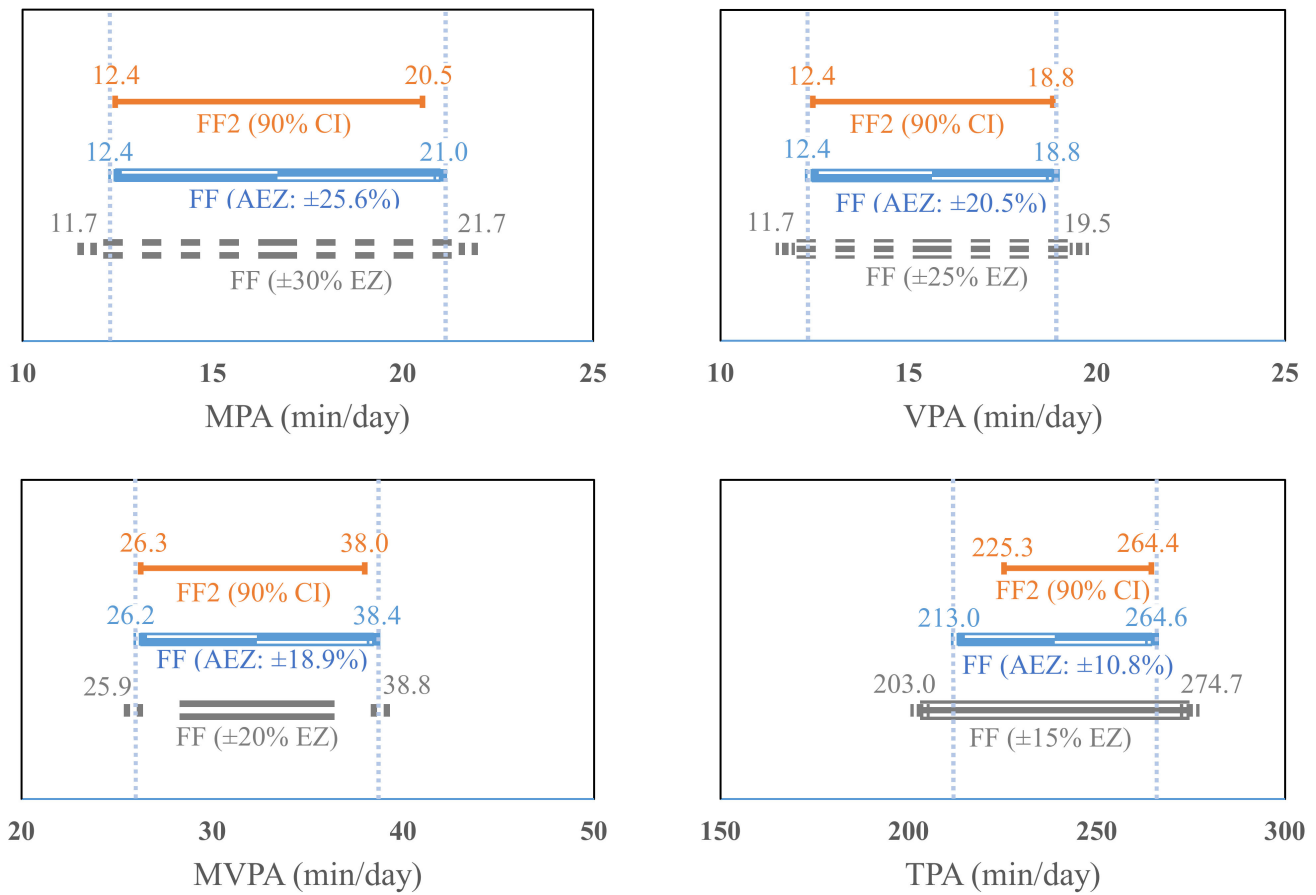
Equivalence testing for SED and PA. CI: Confidence interval; AEZ: Actual equivalence zone; EZ: Equivalence zone; SED: Sedentary behavior; LPA: Light physical activity; MPA: Moderate physical activity; VPA: Vigorous physical activity, MVPA: Moderate-to-vigorous physical activity; TPA: Total physical activity; Vertical dashed lines show the actual bounds within which the FF2 is statistically equivalent to the FF for SED and PA estimates.

Results from the equivalence tests are presented in Figure 3. The calculated 90% confidence interval (CI) of the estimate in SED from the FF2 (90% CI = 778.1 to 884.2 min/day) was within ±10% EZ (10% EZ = 753.5 to 920.9 min/day, actual EZ = ±7.1%) of the SED estimate from the FF. Additionally, the 90% CI in LPA and TPA estimates (LPA = 196.7 to 228.9; TPA = 225.3 to 264.4) from the FF2 fell within ±15% EZ (LPA = 175.5 to 237.5, actual EZ = ±10.9%; TPA = 203 to 274.7, actual EZ = 10.8%) of the estimates from the FF. The EZ of MPA and VPA estimates from the FF were established ± 30% (11.7 to 21.7 min/day, actual EZ = ±25.6%) and ±25% (11.7 to 19.5 min/day, actual EZ = ±20.5%), respectively, which included the 90% CI of the MPA and VPA estimates from the FF2 (12.4 to 20.5; 12.4 to 18.8 min/day). Additionally, the 90% CI of the moderate-to-vigorous PA (MVPA) estimate (26.3 to 28 min/day) from the FF2 fell within ±20% EZ (25.9 to 38.8 min/day, actual EZ = ±18.9%) of the estimate from the FF.

## 4. Discussion

The current study examined the inter-device reliability between the FF and FF2 in estimating time spent in SED and PA during a free-living condition over fourteen days. Overall, our results indicated that the FF2 could provide similar estimates of SED and PA compared to the FF during a free-living condition. More specifically, there was a strong positive relationship, relatively low measurement discords, and no apparent bias for agreement between the FF and FF2 in classifying SED and MVPA in a free-living condition. Previous research has shown strong positive correlations between the FF and research-grade accelerometer in their estimates of SED and MVPA [20]. With the established validity of the FF in mind, our findings of high inter-device agreement between the FF and FF2 also suggest that the FF2 can accurately classify SED and MVPA. Given the frequent updates to Fitbit models, our findings suggest that the FF and FF2 can be interchangeably used to measure SED and MVPA for large cohorts and longitudinal research in measurement and intervention during a free-living setting.

An important finding of this study was that both FF and FF2 could be comparably used as PA measurement tools in research, aiming to investigate time spent in SED and MVPA. The self-monitoring by survey tools could be influenced by the responders’ social desirability and recall bias [21]. For this reason and with advances in wearable technologies, accelerometry-based activity monitors such as Fitbit have emerged as popular device for measuring PA. A few previous studies indicated that the FF can provide relatively accurate SED and MVPA estimates under free-living conditions [20,22,23] A recent study by Redenius et al., reported that the FF yielded similar SED estimates with low measurement error (mean difference = 37 min/day, *p* = 0.21, mean absolute percent error = 6.8%) compared to ActiGraph GT3X+ accelerometer in healthy adults [20]. In addition, other previous studies revealed that the FF overestimated MVPA time by 3% in healthy young adults (age range: 19 to 37) [23] and 10% in older adults (age: 65.6 ± 6.9 years; the rate of cardiac patients: 58.9%) [22] when compared to the ActiGraph accelerometer. Given the recommended acceptable mean errors are within 10% under free-living [24,25] and 20% for clinical purposes [26], the FF would be considered a valid PA measurement tool to assess the amount of MVPA in adults. With time, however, the manufacturer replaced the FF with FF2 for offering more features, such as automatic exercise recognition and call notification, and convenience through enhancements made to existing configurations. Since the specifications within the Fitbit’s activity tracker product line are similar, the agreement between the FF and FF2 is important to increase the internal validity and reliability of measurements in studies using both FF and FF2. In this regard, we found a strong agreement evident between the FF and FF2 for the derived SED and MVPA estimates. Moreover, the strong inter-device agreement would enable harmonized comparisons of PA data across studies using either the FF or FF2 [27]. Therefore, findings on the validity of FF from previous studies and strong correlations and measurement agreements observed in the present study indicated that both FF and FF2 are promising PA measurement tools to estimate time spent in SED and MVPA in PA epidemiological research.

Another important finding was that the FF2 had a systematic bias in estimating LPA in comparison with the FF. More specifically, the FF2 increasingly overestimated LPA compared to the FF as the mean volume of LPA increased (r = −0.4, *p* = 0.02; Figure 2). Additionally, the overall mean bias of the FF2 was 6.3 min/day (95% CI: −11.8 to −0.7) that might lead to the misclassification for SED (Table 2 and Figure 2). It is speculated that either FF or FF2 might capture relatively low or high magnitudes of wrist acceleration for SED or LPA of some participants (e.g., typing or standing) during a free-living condition. Accordingly, the observed difference for the LPA estimate between the FF and FF2 might influence the difference for the SED estimate. However, the mean bias for LPA did not significantly impact the agreement of SED estimates between the FF and FF2 under a free-living condition. It is noteworthy that the equivalence test demonstrated the FF2’s LPA estimate was equivalent to the FF at ±15% equivalence zone (EZ). The equivalence testing provides a direct evaluation for measurement agreement between FF and FF2; 15% to 20% error can be an acceptable error for equivalence depending on the measurement purposes or populations [28]. Moreover, we calculated the actual bounds within which the FF2 is statistically equivalent to the FF for LPA estimates by using the equivalence testing. The calculated actual EZ was ±10.9%, indicating the measurement discord of the FF2 for LPA was less than 10.9% compared to the estimate from the FF. Thus, the FF2 is still comparable to the FF in estimating overall LPA time.

It is noteworthy that the FF underestimated time spent in LPA against a research-grade accelerometer under free-living conditions. The study by Dominick et al., reported that the FF significantly underestimated the daily proportion of LPA time compared to the hip-worn ActiGraph GT3X+ (mean difference: −34.0 ± 3.0%, *p* < 0.001, Cohen effect size = 6.9) in young adults (age range: 19 to 37 years) [23] A study with pregnant women (age range: 32.7 ± 4.2 years) also showed the FF significantly underestimated time spent in LPA (mean difference: 25.6 ± 38.7 min/day, *p* = 0.02) in comparison with ActiGraph GT3X+ [29]. One possible explanation is that the FF may not consider the short bouts of activity by frequent breaks or slower motions as LPA [30]. It is speculated that the proprietary Fitbit algorithms were geared toward capturing the higher bouts of activity. However, it is not known how the Fitbit algorithms calculate time spent in SED and PA and how these algorithms may change over time. Considering our finding that the FF2 increasingly overestimated LPA compared to the FF as mean volume increased, the ability to estimate LPA was likely improved in the FF2. Therefore, it would be valuable to evaluate the validity of FF2 in estimating LPA in future research.

The observed high inter-device agreement of the FF in the current study also underlines that Fitbit can be a PA measurement tool in large-scale prospective studies involving multiple measures throughout follow-up periods. To date, research-grade accelerometers have been widely used to gain insight into activity levels among various populations in a large cohort and longitudinal research, such as the National Health and Nutrition Examination Survey [31]. Unlike the research-grade accelerometer, Fitbit frequently releases new devices on the commercial market, replacing older models. In other words, it can be unavoidable to include varying models of Fitbit in a longitudinal study. In this regard, Fitbit devices could be utilized extensively to measure PA in longitudinal studies, regardless of the model, if the inter-device agreement is established across Fitbit models.

The current study found that there was a high inter-device agreement between Fitbit models with similar specifications. Additionally, this finding is consistent with a previous systematic review that high inter-device agreement for PA measurements between Fitbit models [32]. Accordingly, the use of varying Fitbit models as PA measurement tools may be feasible in longitudinal studies. Moreover, allowing participants to choose a preferred model of Fitbit would be more practical and better in terms of usability and adherence to wearing the device for a long-term period. Supporting this, a recent large cohort study called All of Us Research Program measures PA characteristics of the participants who use their own Fitbit device without restricting a particular model of the device. In light of the established high inter-device agreement, the current study could support the idea that the use of various Fitbit devices is feasible for tracking PA characteristics in large-scale longitudinal studies.

Furthermore, most Fitbit devices are relatively inexpensive, simple, and convenient compared to research-grade accelerometers. Additionally, nearly all Fitbit devices are popular to varying age groups and worn on the wrist, which improves participants’ adherence to wearing time over prolonged periods [33]. These characteristics, along with the FF’s high inter-model agreement observed in this study, support that Fitbit devices show excellent potential for use in intervention studies that may require high compliance of wearing activity monitors as either an intervention or a measurement tool, regardless of model generations. Additionally, many researchers and clinicians want to obtain the data ready for analysis directly from the activity tracker. In this regard, Fitbit offers a relatively straightforward web-based database/dashboard to aggregate and process data from the FF and FF2 without additional data processing. In light of the established inter-device agreement and extended advantages, the FF and FF2 can be a good choice for use in clinical studies, aiming to assess activity intensity that occurs all day for a long-term duration.

This study has several strengths that include assessing PA behaviors for 14 consecutive days and the high adherence to wearing time (Table 1). Most previous studies have assessed SED and PA estimates using Fitbit devices under a free-living condition for 2 to 7 days [6,8,20,23]. The longer duration of the measurement is critical for determining the reliability of PA measurement because it would more likely cover a variety of activity patterns, which may have enabled a more valid test of the inter-model agreement between the FF and FF2. Additionally, our participants were asked to wear the FF and FF2 all day, except for charging and aquatic activities, for the rigorous assessment. As a result, the observed average wear time was 1074 ± 174 min/day (Table 1). The minimum burden of wearing the light-weighted devices might enable high adherence to wearing time in our participants [33,34]. Consequently, the current study was able to obtain reliable data regarding the inter-device agreement between the FF and FF2. This study is not without limitations. The generalizability of the findings from the current study could be limited due to the relatively small size and homogenous characteristics of our sample.

## 5. Conclusions

In conclusion, the Fitbit Flex 2 (FF2) is comparable to the Fitbit Flex (FF) in its ability to estimate SED and MVPA. However, there was not a strong agreement between the FF and FF2 for LPA estimate. The FF is a valid and reliable activity monitor and has been widely used in estimating SED and MVPA and promoting PA across all ages and populations. Although the FF is a valid activity monitor in estimating SED and MVPA, Fitbit’s frequent product updates may limit its utilization in longitudinal studies. In our study, the observed agreement between the FF and FF2 indicates little to no significant difference for SED and MVPA estimates between model generations. Thus, both FF and FF2 can concurrently be used, or the predecessor of Fitbit could be replaced with newer models of Fitbit devices without compromising the validity of SED and MVPA measurement in observational and clinical research.

## Figures and Tables

**Table 1:** Characteristics of study participant by gender, Mean (SD) or percent.

Characteristics	Total (*N* = 33)	Male (*N* = 11)	Female (*N* = 22)
Age (years)	23.9 (0.8)	24.1 (0.8)	23.8 (0.8)
Race (%)			
White	94% (*n* = 31)	91% (*n* = 10)	95% (*n* = 21)
Other	6% (*n* = 2)	9% (*n* = 1)	5% (*n* = 1)
Weight (kg)	74.8 (15.4)	91.3 (14.6)	66.6 (6.9)
Height (cm)	174.8 (8.2)	181.3 (8.1)	171.6 (6.2)
BMI (kg/m^2^)	24.3 (3.7)	27.8 (3.9)	22.6 (2.2)
Wear time (min/day)	1074 (174)	1116 (210)	1056 (174)

**Table 2:** Mean differences and Mean Absolute Percent Errors (MAPE) of SED and PA between FF and FF2.

Intensity	FF (SD)	FF2 (SD)	Mean Difference (SD)	95% CI	MAPE
SED	837.2 min (183.3)	831.1 min (179.9)	6.0 min (18.9)	−0.7 to 12.7	0.7%
LPA	206.5 min (48.4)	212.8 min (54.6)	−6.3 min (15.6)	−11.8 to −0.7	3.1%
MPA	16.7 min (13.1)	16.5 min (13.7)	0.2 min (2.6)	−0.7 to 1.2	1.2%
VPA	15.6 min (10.9)	15.6 min (10.8)	−0.01 min (2.9)	−1.0 to 1.0	0.1%
MVPA	32.3 min (19.3)	32.1 min (19.9)	0.2 min (4.7)	−1.5 to 1.9	0.6%
TPA	238.8 min (59.4)	244.9 min (66.4)	−6.0 min (18.9)	−12.7 to 0.7	2.5%

SD: Standard deviation; CI: Confidence interval; SED: Sedentary behavior; LPA: Light physical activity; MPA: Moderate physical activity; VPA: Vigorous physical activity, MVPA: Moderate-to-vigorous physical activity; TPA: Total physical activity.

## Data Availability

The datasets of the current study are available from the authors on reasonable request.
